# Overlooked Impact of Moisture on the Stability of Printing Ink and Its Impact on Recycled Low-Density Polyethylene (LDPE) Quality

**DOI:** 10.3390/polym16233234

**Published:** 2024-11-21

**Authors:** Jinyang Guo, Willi Wagner, Iryna Atamaniuk, Zhi Kai Chong, Ayah Alassali, Kerstin Kuchta

**Affiliations:** Circular Resource Engineering and Management (CREM), Hamburg University of Technology (TUHH), Blohm Str. 15, 21079 Hamburg, Germanykuchta@tuhh.de (K.K.)

**Keywords:** plastic recycling, printing ink, design for recycling, polymer degradation

## Abstract

Printing inks, composed of binders, pigments, and additives, are essential components in plastic packaging but complicate recycling due to plastic contamination and degradation. While polyolefins are resistant to hydrolytic degradation, moisture generated from upstream cleaning processes, which is often ignored, can accelerate the degradation of ink binders, affecting the recyclate quality. This study has examined the impact of 3 wt.% moisture, introduced before extrusion, on the degradation of nitrocellulose (NC), polyurethane (PU), polyvinyl butyral (PVB), and cellulose acetate propionate (CAP) binders mixed with virgin, low-density polyethylene (LDPE) at varying concentrations to simulate contamination levels. Control samples were prepared by extrusion under dry conditions and using p-xylene to compare with degradation-free conditions. Analyses, including the measurement of the melt–flow index (MFI), tensile testing, FTIR (Fourier transform infrared spectroscopy), TGA (thermogravimetry analysis), and gas chromatography mass spectroscopy (GC-MS) have established that NC is fully degraded, causing discoloration and altering the MFI. Moreover, PU degrades mainly in the presence of moisture, contrary to previous findings. In contrast, PVB does not degrade but exhibits modified mechanical properties; whereas, CAP shows minimal impact. The findings of this research demonstrate the critical role of moisture in determining recyclability, informing strategies for ink selection and recycling processes to facilitate plastic packaging circularity.

## 1. Introduction

In the European Union (EU), annual plastic consumption reaches approximately 50 million metric tons, with nearly 40% used in packaging [1] having a service life of 0.5–1 year [2]. As the predominant form of packaging, 5 million tons of single-layer, flexible plastic packaging (75% from low-density polyethylene (LDPE) and 25% from polypropylene (PP)) are converted into household packaging that includes food and a range of commercial products [3].

Mechanical recycling is the preferred end-of-life option for plastic packaging waste, contributing to CO_2_ emissions reduction and the conservation of fossil resources [4,5]. This waste is collected and sorted on the basis of physical properties (primarily using wind shifters and ballistic separators) and polymer types (using near-infrared spectroscopy) and, then, transported as pressed bales to recycling plants [6,7]. The materials undergo shredding, washing, drying, and extrusion to produce recycled plastic, primarily as granulates [8,9]. Currently, the recycling rate of single-layer, flexible plastic packaging is approximately 30% (net converted to recyclate), with only 10% achieving circularity in comparable packaging applications [3]. There is a pressing need for alternative technical options in packaging design and cleaning technologies directed at improving the quality of recycled plastics.

### 1.1. Printing Ink for Plastic Packaging

Printing ink is a necessary component in modern packaging for conveying product information and for marketing purposes [10]. Printing ink comprises pigments, binder resins (for adhesion, flexibility, and resistance), and various additives, typically suspended in organic solutions (alcohols or esters) [11,12]. During the drying process employed in industrial plastic packaging label printing, the solvent is evaporated; whereas, solvent-free inks are cured using ultraviolet radiation [13]. The composition of standard printing inks used in plastic packaging is provided in the Supplementary Material (S1).

Nitrocellulose (NC) is the dominant binder type in current post-consumer, flexible packaging streams, with an 80% market share in Europe, the Middle East, and Africa [12]. Thermal degradation of NC is initiated at 160 °C [14], with appreciable degradation at typical extrusion temperatures (180–260 °C), generating noxious gases and a brownish discoloration in the recycled product [15,16].

Polyurethane (PU) represents another binder option for printing on plastic packaging [12]. PU, particularly aliphatic PUs, is thermally stable below 250 °C based on reported thermal gravimetry analysis (TGA) [17,18]. However, as a polycondensated polymer, it undergoes hydrolytic degradation at lower temperatures that coincide with the extrusion used in LDPE recycling [19].

Polyvinyl butyral (PVB) is a less commonly used binder. A previous study has shown that de-inking PVB requires higher temperatures and more alkaline conditions [12]. PVB is characterized by a high thermal stability, where degradation requires temperatures in excess of 260 °C [20,21], and the polymer C-C backbone is resistant to hydrolytic degradation [22,23].

Cellulose acetate propionate (CAP) is widely used in food packaging [12]. Although the CAP binder content in recycling streams is low (<1 wt.%), its inclusion in the sample matrix is significant due to a high associated resistance to de-inking and thermal stability (up to 300 °C) [24,25].

An overview of current printing ink binders is presented in Table 1.

### 1.2. Printing Ink Contamination

Contamination by printing ink present in the recycled plastic was first reported by Gecol et al. 2001. This study compared the quality of different post-consumer, flexible plastic packaging and identified agglomerates of printing ink by scanning electromagnetic microscopy (SEM) [33].

The contamination caused by printing ink in the recycling process has led to various studies investigating methods to remove the ink before extrusion. Initial studies by Gecol et al. concluded that use of cetyltrimethylammonium bromide (CTAB) as a cationic surfactant was the most effective means of removing printing ink at pH 13 [33,34]. Ügdüler et al. 2023 [28] examined the “de-inkability” of different printing ink systems and considered various organic and inorganic solutions, such as acetone, ethyl acetate, formic acid, sulfuric acid, NaOH, and CTAB solution. The removal of PU-based printing ink by gamma-valerolactone has been assessed in treating the polyethylene terephthalate (PET) layer of multilayered plastic packaging as part of a solvent-based recycling process [35]. Lisiecki et al. [16] carried out a recent study comparing the impact of NC and PU binders on the quality of mechanically recycled plastic, with and without different pigments. The NC and PU printing inks lowered the quality of the recycled LDPE, which was within the acceptable range for the film blowing process. The presence of printing ink pigments has a greater effect in lowering the quality of recycled plastic [16]. Guo et al. 2024 reviewed the stability of different printing ink binders in thermal and hydrolytic degradation. They concluded that humidity can alter the degradation of printing ink binders in the extrusion process [36]. Humidity (3–5 wt.%) may be introduced during the washing process, where residual water is present in the washed flakes after drying [37].

### 1.3. Objectives of This Study

Based on an overview of the current literature presented in Section 1.2, it is concluded that printing ink associated with plastic packaging is a source of contamination that enters the polymer matrix of recycled plastic at an early stage in the life cycle. The following knowledge gaps still exist and are addressed in this work:

(1) The influence of residual humidity after the drying process on degradation behavior has been identified as a contributing factor [36], but there is a lack of experimental evidence to support this;
(2) The impact of the printing ink binder on the quality of recycled plastics remains unclear and warrants further investigation;
(3) (3) Having established the degradation behavior and associated effects on recycled plastic quality, this information should be directed at optimizing packaging design and recycling processes to work effectively in tandem.

## 2. Materials and Methods

**Figure 1:**
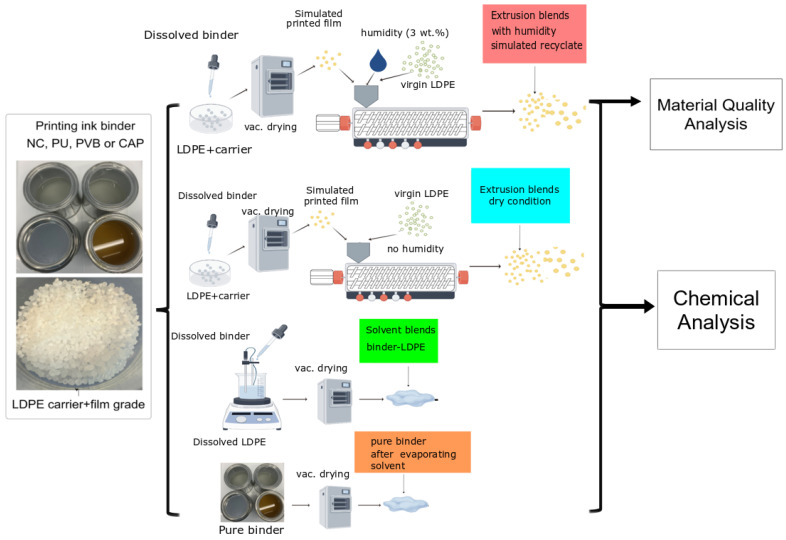
Schematic representation of the overall experimental approach.

The overall experimental approach is illustrated in Figure 1. Two different groups of printing ink binder–LDPE blends were prepared. Extrusion blends were used to simulate the recycled printed LDPE generated in an industrial recycling process. The other group of samples included solvent blends, extrusion blends without humidity, and pure binder in solid form, which served as a baseline, enabling a meaningful comparison of degradation caused by extrusion processes with that induced by moisture.

All the printing ink binders were provided by Siegwerk Druckfarben AG and Co.KGaA, Siegburg, Germany, and delivered dissolved in organic solutions. Details regarding the binders are given in the Supplementary Material (S2.1). The binder samples employed in this study include nitrocellulose (NC), aliphatic polyurethane (PU), polyvinyl butyral (PVB), and barrier binder cellulose acetate propionate (CAP). The use of non-pigmented, clear varnish samples enabled an effective analysis of the impact of the binders, circumventing the effects due to pigments reported in prior studies.

The first group of blends (Table 2a) was prepared for investigating the degradation behavior of the printing ink binder resins. Each binder sample (NC, PU, PVB, and CAP) was blended with LDPE using both extrusion and solvent blend methods. For the NC and PU binders, the extrusion blends were prepared both with 3 wt.% humidity and dry conditions, as PVB and CAP are reported to be stable against thermal and hydrolysis degradation under extrusion temperature, as shown in Table 1. In addition, the four binder samples were vacuum dried and used as comparative references. The samples shown in Table 2a were subjected to FTIR and thermogravimetry (TGA) analysis, to investigate whether humidity facilitates degradation during the extrusion process.

The second group of blends (Table 2b) was prepared by mixing the dissolved printing ink binder resin granulated with LPDE at room temperature, followed by vacuum drying, to simulate the printing process on the LDPE film. After drying, the binder–LDPE mixtures were subjected to extrusion that simulated recycling. Humidity was introduced during extrusion to mimic the humidity that is a feature of industrial recycling. The concentration of binder in LDPE was set at a gradient to simulate the varying printing ink content in the recycling input stream. Material quality analysis was conducted only on these samples extruded with humidity, since these samples simulated the recyclate from the current industrial recycling process.

An overview of all the samples prepared in this study is provided in Table 2a,b.

### 2.1. Sample Preparation

Each binder resin sample was blended with 20 g carrier–LDPE (Evonik Accurel XP400, Essen, Germany) using a 10 mL syringe injector to ensure homogenized mixing during the extrusion process, simulating the recycling of printed LDPE films. Concentrations were varied in gradients of 1, 3, and 5% for NC, PU, and PVB samples, simulating light-printed (post-consumer waste), mixed, and heavy (full area)-printed (post-industrial) material streams. Given the limited application of CAP, the gradient was set at 0.5 and 1%. The syringe injector was replaced when switching between different types of binder to avoid cross-contamination. As the binder resins were supplied in solvents, the LDPE–binder resin mixtures were first placed in an evaporation dish and subjected to solvent evaporation in a vacuum oven at 60 °C for 24 h.

Following evaporation, 75 g LDPE granulates (INEOS 23H430, Cologne, Germany) were mixed with the prepared binder resins for extrusion. In order to mimic residual moisture from the pre-extrusion washing process, 3 g distilled water was carefully added to each sample before it entered the barrel of the extruder, which was not needed for the samples NC-5D and PU-5D. A double-screw extruder (16/25 D/L) (Thermo Fisher Scientific, Karlsruhe, Germany) was used in the extrusion process, with the temperature profile 165-180-195-210-230 °C, covering the extrusion temperature range for LDPE recycling [9]. Details of the sample matrix and preparation procedure are provided in the Supplementary Material (S2.2).

The degradation of the binder resin during extrusion was investigated using mixtures of LDPE/carrier resin and 5 wt.% binders dissolved in xylene at 70 °C under a nitrogen atmosphere; 3.75 g LDPE granules and 1 g LDPE carrier resin were mixed with 20 g p-xylene and heated to 70 °C in nitrogen until the LDPE was completely dissolved. The heating was discontinued, and a 5 wt.% dry content equivalent of binder was added to the dissolved LDPE. The sample was then dried in a vacuum oven at 0.1 bar and 60 °C for 24 h to completely evaporate the organic solution. Details of the sample matrix and preparation procedure are provided in the Supplementary Material (S2.3).

### 2.2. Material Quality Assessment

#### 2.2.1. Melt–Flow Index

The processability of the prepared blends was evaluated by determining the melt–flow index using a ZwickRoell Mflow melt–flow indexer (Zwick & Roell, Ulm, Germany), applying ISO 1133-1 standard test methods [38]. The testing conditions were set at 190 °C with a 2.16 kg load over 10 min. To ensure accuracy, the software conducted three replications per test, and to further mitigate potential errors, each sample underwent three separate tests.

#### 2.2.2. Mechanical Properties

In tensile strength tests, samples were prepared using an injection molding machine (Babyplast 6/10, Milan, Italy), in accordance with ISO 294-1 type 5A standards [39]. Tensile strength assessments were conducted on a universal testing machine (Darto PM3, Darto GmbH, Wuppertal, Germany); six samples were tested for each blend.

#### 2.2.3. Independent *t*-Test

The analysis data revealed small differences in the values obtained for different samples. In order to determine if the differences in each parameter were significant, a *t*-test was carried out on all samples and related to the blank sample and other samples with the same binder mixed with different concentrations, using Equation (1). A *p*-value was computed to evaluate the significance of the *t*-value against the null hypothesis (assuming the values have no difference) [40].

(1) t=(X1¯−X2¯)s12n1+s22n2

where:

*X*: mean value of the parameter;

*s*: square of the standard deviation;

*n*: sample size.

The critical t-value was obtained from a t-distribution table, corresponding to the calculated degrees of freedom and a significance level (α = 0.05). The calculated *t*-value from a comparison of two samples was assessed in terms of the critical t-value to determine the statistical significance.

The degree of freedom for the *t*-test was calculated using Equation (2).

(2) $$df=n_{1}+n_{2}-2$$

where:

*df*: degree of freedom;

*n*: sample size.

For a two-tailed p-test, the *p*-value is calculated using Equation (3).

(3) $$p=2P(T>abs.\left(t\right))$$

where:

*p*: probability of obtaining a t-value greater than the observed value under the null hypothesis, where a threshold of 0.05 is applied;

*T*: theoretical t-distribution for a given number of degrees of freedom.

### 2.3. Chemical Analysis

#### 2.3.1. Fourier Transform Infrared Spectroscopy (FTIR)

Fourier transform infrared (FTIR) spectroscopy was employed to assess potential polymeric degradation and to identify the functional groups associated with the binders in the LDPE granulates. This analysis was carried out using a Bruker Vertex 70 FTIR spectrometer (Bruker Corporation, Billerica, MA, USA), covering a wavelength range of 4000 to 800 cm^−1^, with 32 scans and a resolution of 4 cm^−1^. The acquired spectral data were analyzed using OPUS 7.2 software. Scanning was conducted in triplicate, and the average spectra calculated following baseline normalization.

The spectra of the dried pure binder resin, the solvent blend, and the extrusion blends were overlaid and compared. Specific peaks associated with functional groups in the binder resin were identified from the pure binder spectra. Changes in peak intensity in the solvent and extrusion blends were analyzed to determine the extent of degradation. The reduction in or disappearance of characteristic peaks in the extrusion blend relative to the solvent blend and pure binder served to indicate binder resin degradation during extrusions.

#### 2.3.2. Thermogravimetry Analysis (TGA)

Each 0.5 g sample described in Table 2a was subjected to thermogravimetric analysis using a Linseis TGA 1000 analyzer (Linseis Messgeraete GmbH, Selb, Germany). The analysis was conducted in the temperature range of 20–600 °C, with a heating rate of 5 °C/min under a nitrogen atmosphere.

#### 2.3.3. Screening Potential Degradation Products by GC-MS

In order to evaluate the effect of trace substances, such as printing ink additives and degradation products, analysis by gas chromatography combined with mass spectroscopy (GC-MS) was carried out.

All the test materials were cut into samples of approximately 4 mm size; 0.4 g samples were treated with 98% ethanol as a suitable polar extraction solvent [36]. The extraction was carried out at 20 °C for 10 days. In each extraction set, a blank sample with organic solution (ethanol, HPLC grade, Carl Roth) was prepared. The extract was concentrated in a nitrogen atmosphere to 0.3 mL for subsequent GC-MS analysis.

The GC-MS analyses were conducted using an Agilent 8860 GC system (Agilent Technologies, Santa Clara, CA, USA) equipped with a single quadrupole MSD (Agilent 5977B) and an HP-5 MS column (30 m × 0.25 mm i.d., 0.25 μm film thickness). The system was controlled by a GC-MSD MassHunter with MSD ChemStation DA, Version 10.0. Helium served as the carrier gas.

The screening was conducted using Mass Hunter Unknowns Analysis (ver.10.1, Agilent Technologies), coupled with the NIST 20 Library for compound identification.

## 3. Results

### 3.1. Degradation by Extrusion Under Humid Conditions

#### 3.1.1. NC Degradation

**Figure 2:**
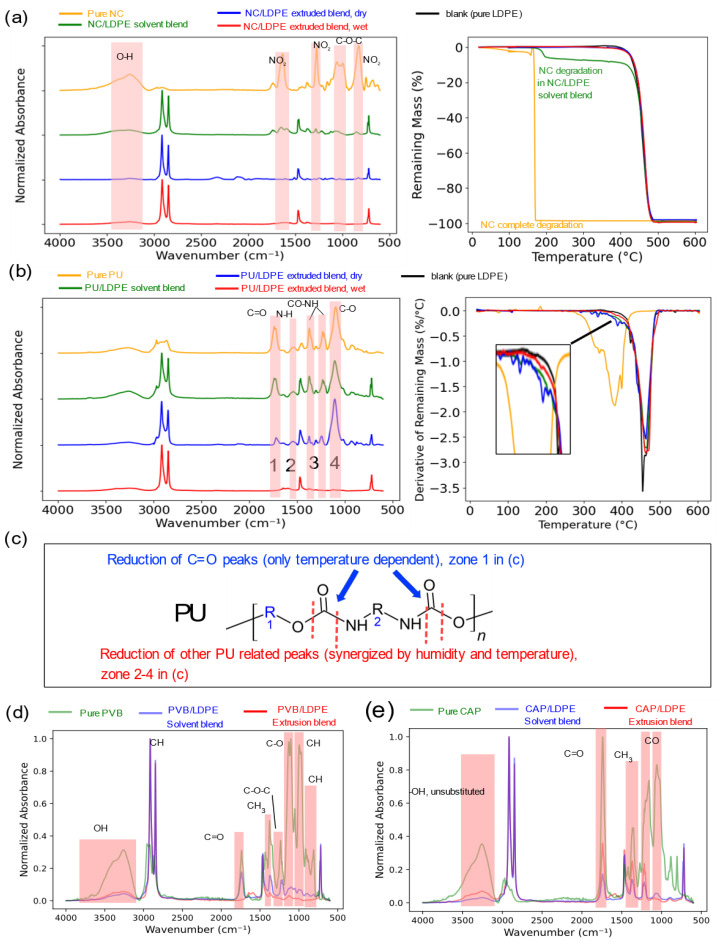
(**a**) **left**: FTIR spectra pure NC (orange), NC/LDPE solvent blend (green), NC/LDPE extrusion blend without humidity (blue), and NC/LDPE extrusion blend with humidity(red); **right**: TGA curves of these NC-containing samples, compared with pure LDPE (black). (**b**) **left**: FTIR spectra pure PU (orange), PU/LDPE solvent blend (green), PU/LDPE extrusion blend without humidity (blue), and PU/LDPE extrusion blend with humidity(red); spectral zones are highlighted as follows: 1 (C=O), 2 (N-H), 3 (C-N (CO-NH)), and 4 (C-O). **right**: DTG curves (derived from TGA data) of all PU-containing samples, compared with pure LDPE (black profile) for clearer determination of PU behavior in LDPE. (**c**) Hydrolysis of PU accounting for peak intensity differences between the PU/LDPE solvent blend and the extrusion blend based on the hydrolysis degradation mechanism [43]. (**d**) FTIR spectra of pure PVB (green profile) after drying, 5 wt.% PVB/LDPE solvent blend (blue profile), 5 wt.% PVB/LDPE extrusion blend (red profile) with spectra assignments according to Moudam and Lakbita 2021 [44]. (**e**) FTIR spectra of pure CAP (green profile) after drying process, 1 wt.% CAP/LDPE solvent blend (blue profile), and 1 wt.% CAP/LDPE extrusion blend (red profile) with spectra assignments [42,45].

In the FTIR analysis (Figure 2a, left) of pure NC (orange profile), the NC/LDPE solvent blend (green profile), the NC/LDPE extrusion blend without humidity (blue profile), and the NC/LDPE extrusion blend with humidity (red profile), it was observed that the NC/LDPE solvent blend retained all the NC characteristic peaks in the highlighted zones, notably the -ONO_2_ groups at 1660–1625 cm^−1^, 1280–1270 cm^−1^, and 870–830 cm^−1^ [41,42]. These peaks were absent in both NC/LDPE extrusion blends, regardless of the presence of humidity. In the wavelength range 3300–3100 cm^−1^, representing overlapping -OH and -NH regions, both NC/LDPE extrusion blends (blue and red profile) showed significant lower peak intensities than the NC/LDPE solvent blend (green profile).

Thermogravimetric analysis (TGA) results (Figure 2a, right) revealed that pure NC (orange profile) underwent complete degradation at approximately 180 °C, consistent with previous findings summarized in Table 1. The NC solvent blend (green profile) displayed a similar degradation profile of the NC part in LDPE, with completion at around 200 °C. In contrast, both extrusion blends, with and without humidity, showed TGA profiles similar to that of the pure LDPE sample. The combined FTIR spectroscopy and TGA results confirm the degradation of NC during LDPE extrusion, occurring within the temperature range of 180–200 °C, irrespective of the presence of moisture.

#### 3.1.2. PU Degradation

The FTIR spectra of pure PU binder (orange profile), the PU/LDPE solvent blend (green profile), the PU/LDPE extrusion blend without humidity (blue profile), and the PU/LDPE extrusion blend with humidity (red profile) (Figure 2b, left) revealed distinct changes indicative of degradation. The PU/LDPE solvent blend retained all the characteristic peaks of the pure PU binder, as highlighted in Zones 1–4 in Figure 2b, left. These include C=O stretching (1738–1724 cm^−1^), N-H bending (1538 cm^−1^), -NH-CO stretching (1400–1350 cm^−1^ and 1230–1217 cm^−1^), and C-O stretching (1095 cm^−1^). The PU/LDPE extrusion blend without humidity preserved these PU-related peaks, despite the reduced intensity of the C=O stretching peak (1738–1724 cm^−1^), consistent with previous studies reporting partial C=O group degradation in PU below 200 °C [17,46]. However, the PU/LDPE extrusion blend with humidity exhibited a significant decrease in the intensity of all PU-related peaks, as highlighted in Figure 2b, left.

The differential thermogravimetry (DTG) curves of all PU-related samples, derived from their TGA curves, are shown in Figure 2b, right. The pure PU binder (orange profile) began thermal degradation at 250 °C, completing at approximately 420 °C. This behavior suggests that the PU binder maintains its polymeric structure in the PU/LDPE extrusion blend without humidity. The DTG curve of this sample (blue profile in Figure 2b, right) displayed several small peaks at 320–350 °C, closely resembling the profile of pure PU, confirming the presence of small amounts of PU in the blend. In contrast, the PU/LDPE extrusion blend with humidity showed a decomposition profile closely aligned with that of pure LDPE (black profile), characterized by a single sharp degradation peak at 420–480 °C.

The combined FTIR and TGA analyses confirm the hypothesis that PU binder degradation is synergistically driven by humidity and extrusion temperature, as illustrated in Figure 2c. While the degradation of the C=O bond (Zone 1 in Figure 2b, left) is only dependent on the processing temperature, other changes in the FTIR spectra correspond to the scission of urethane linkages and the formation of degradation products, consistent with the reduced peak intensities observed in Zones 2–4 of Figure 2b, left. The hydrolytic PU degradation products can include diamines, polyols, and CO_2_ [43]. Another factor contributing to polymer degradation is oxidation-induced degradation [17,47]. During the extrusion process, the extruder barrel was filled with molten polymer, and the process design ensured effective sealing, minimizing exposure to external oxygen or ambient air.

#### 3.1.3. PVB and CAP Degradation

A comparison of the FTIR spectra (Figure 2d) for pure PVB, the PVB/LDPE solvent blend, and the PVB/LDPE extrusion blend reveals a reduction in absorption intensity of the C-O-C and C=O bonds. This response may be related to a PVB side chain degradation caused by the extrusion temperature [48]. However, the current literature does not provide a detailed description of the PVB degradation mechanism for extrusion in the presence of moisture [21,49]. It is not possible to conclusively determine whether the degradation is confined to the side chain due to the background contribution of LDPE in the TGA analysis due to the low mass change. Nevertheless, given the stability of the C-C single bond, significant degradation is not expected under typical extrusion conditions [20].

In the case of CAP, no noticeable changes were observed in the FTIR spectra for the solvent and extrusion blends (Figure 2e). Minor differences in the C=O (1739 cm^−1^) and C-O (1200–1160 cm^−1^) signals may indicate some side chain reactions, a feature that requires further detailed investigation.

### 3.2. Quality of Simulated Recyclates with Binder

#### 3.2.1. Impact of Moisture on Recycled LDPE Quality

In order to isolate the impact of moisture in the extrusion process, different quality parameters associated with the blank sample extruded without moisture (blank dry) and with moisture (blank wet) are presented in Table 3. The quality parameters for the blank wet sample can serve as the benchmark for further comparisons. No statistically significant difference can be determined from all the quality parameters, including MFI, mechanical properties (Young’s modulus, tensile strength, and strain at break), and thermal analysis (crystallization temperature, melt temperature, and degree of crystallinity). This statistical analysis confirms that moisture has no effect on the LDPE quality. Therefore, the quality parameters obtained from the blank wet sample were selected as a suitable benchmark.

#### 3.2.2. Results of Melt–Flow Index

**Figure 3:**
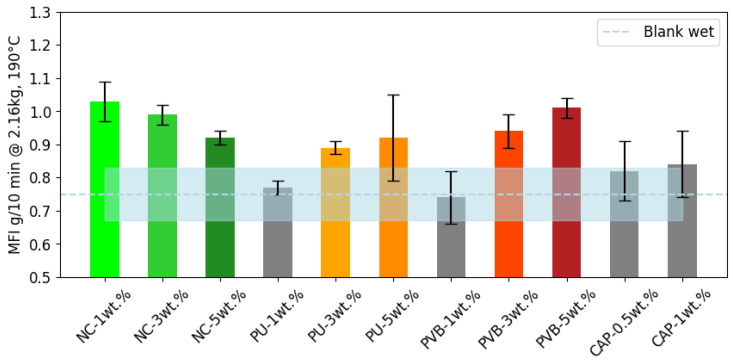
The melt–flow index (MFI) of all the binder–LDPE blends. The blue dotted line represents the benchmark MFI of the blank sample without binder resin and with 3 wt.% water added (blank wet). The area in shadow around the benchmarks represents the standard deviation. The colored bars indicate a statistical difference relative to the blank sample; whereas, the gray bars exhibit no statistically significant difference from the blank sample.

The melt–flow index (MFI) values for different samples are presented in Figure 3. In the case of the NC binders, an initial increase in the MFI of LDPE is observed, presumably influenced by the small molecular, non-phthalate-based plasticizer (citrate esters) in the NC printing ink binder [16,50]. This observed MFI increase is supported by the GC-MS analysis, which detected the presence of tributyl acetylcitrate and hexanedioic acid, bis (2-ethylhexyl) ester that have been reported as plasticizers [51,52]. Notably, these compounds are not degradation byproducts of NC, as they were also detected in the NC/LDPE solvent blend. These findings confirm that plasticizers are intentionally added to NC-based printing inks and contribute to the observed MFI changes. At 5 wt.% NC, a slight reduction in MFI may be attributed to the particles associated with non-volatile degradation products [53,54].

In the case of the PU binder, no significant difference in MFI was observed for the blend with 1 wt.% PU, suggesting a viable processing of printed LDPE with less than 1 wt.% binder to deliver quality comparable with unprinted LDPE. The MFI values for 3 and 5 wt. % PU/LDPE blends were 0.89 ± 0.02 and 0.92 ± 0.13 g/10 min at 190 °C and 2.16 kg, respectively, indicating that there was no statistically significant difference in the PU-3 and PU-5 samples. The increased MFI in PU/LDPE extrusion blends may result from the plasticizing effect of small molecular degradation products from PU or the relatively low molecular weight of undegraded PU. However, the absence or reduction in the intensity of characteristic PU peaks in the FTIR spectra of the PU/LDPE extrusion blend with humidity (Figure 2b left), supported by the DTG result of this sample (red profile in Figure 2b, right), as discussed in Section 3.1.2, confirms PU degradation. The PU/LDPE extrusion blends exhibited progressively higher MFI values with increasing PU concentrations, while cross-linking, if present, would be expected to reduce MFI. This suggests that no significant reaction occurred between -NCO and LDPE, and the free diisocyanates detected by GC-MS likely acted as plasticizers.

No PU degradation products, such as amines or polyols, were detected in the GC-MS analysis. This may be due to their volatility, leading to evaporation or further degradation during extrusion or sample preparation and concentration for GC-MS. Detailed GC-MS results are provided in the Supplementary Material (S3.2). Additionally, the volatility of PU degradation products could create voids within the LDPE polymer matrix or reduce intermolecular friction, thereby increasing LDPE chain mobility and contributing to the observed MFI increase [55,56].

The sample with 1 wt.% PVB showed no significant difference compared with the blank. However, the PVB-3 and PVB-5 exhibited increases in MFI to 0.94 ± 0.05 and 1.01 ± 0.03 g/10 min at 190 °C and 2.16 kg, respectively. This increase in MFI is attributed to the extremely high melting viscosity of pure PVB, which exceeds the measurement limit of the method used for LDPE. During preheating, the flow of vacuum-dried PVB binder from the piston of the melt–flow indexer occurred before the measurement could begin, indicating an exceptionally high MFI. This behavior suggests that the high intrinsic PVB viscosity plays a significant role in increasing the MFI of the blends.

The CAP binder had no statistically significant influence on the melt–flow behavior of the recycled LDPE.

#### 3.2.3. Results of Mechanical Properties

**Figure 4:**
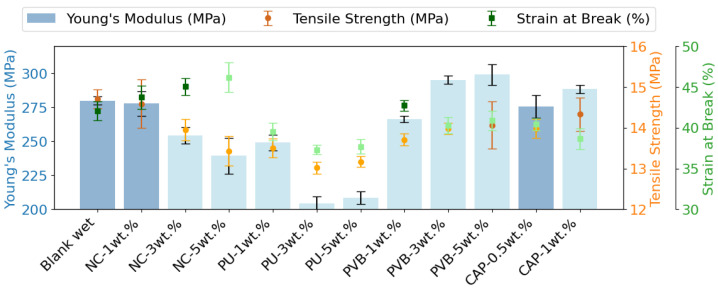
Young’s modulus, tensile strength, and strain at break of all the extruded samples. The bars and darker solid circles indicate no statistical difference compared with the blank sample (blank wet), detailed stress-strain curves are presented in Supplementary Material S3.1.

The tensile properties of LDPE with the NC binder showed a general decrease with increasing NC concentration (Figure 4). At 1 wt. % NC, the Young’s modulus was nearly equivalent to that of virgin polymers (277.83 ± 9.07 MPa compared with 279.83 ± 3.00 MPa for the blank wet sample). However, a continuous decrease in the modulus as NC concentration increased (3 wt.%) to 254.44 ± 6.08 MPa and to 239.40 ± 13.08 MPa at 5 wt.% NC was observed. The tensile strength followed a comparable decreasing trend, while the strain at break increased at higher NC concentrations. These results suggest that the plasticizer in the NC binder increases the elasticity (plasticizing effect) and reduces the stiffness of the LDPE recyclates.

In the case of the PU/LDPE extrusion blends, the Young’s modulus decreased as the PU concentration increased from 1 to 5 wt.%. At 5 wt.% PU, the modulus (208.68 ± 4.60 MPa) was similar to the 3 wt.% sample (204.41 ± 5.21 MPa), indicating a significant decrease relative to the blank wet sample. This reduction may be due to voids in the polymer matrix caused by volatile PU degradation products. The tensile strength and strain at break showed a slight variation (Figure 4).

Mechanical testing of the PVB/LDPE blends have indicated a consistent tensile strength and strain at break across the range of PVB concentrations. However, a noticeable increase in Young’s modulus was observed from 266.49 ± 2.3 MPa for the 1 wt.% blend to 295.10 ± 3.03 MPa and 299.20 ± 7.67 MPa for the 3 wt.% and 5 wt.% blends, respectively. This finding is in agreement with previously reported data for blending PVB with PP at higher (10 and 15 wt.%) concentrations, confirming that PVB functions as an impact modifier (toughener) when blended with polyolefins [57].

The tensile properties of the CAP samples showed a slight change, with an increase in Young’s modulus from 273.53 ± 8.42 MPa to 288.4 ± 2.89 MPa and tensile strength from 13.98 ± 0.23 MPa to 14.33 ± 0.41 MPa when the CAP binder concentration was raised from 0.5 to 1 wt.%, with a slight decrease in the strain at break from 40.43 ± 0.72% to 38.64 ± 1.30%. This is mainly due to the low concentration (up to 1 wt.%) of CAP in the flexible plastic packaging waste stream, as it primarily serves as a barrier layer for packaging material [12].

### 3.3. Overall Impact on Recycled LDPE Quality

Applying the simulated mechanical recycling process and subsequent quality analysis, the overall impact of printing ink binders on the quality of recycled LDPE is summarized in Table 4. In the case of lightly printed material streams (1 wt.% binder, corresponding to less than 2 wt.% printing ink), no significant effect on the recycled LDPE melt–flow properties was observed for PU, PVB, or CAP binders. However, the MFI for NC-based printing inks increased relative to virgin LDPE, which may be attributed to the presence of plasticizers.

In the samples with a higher printing ink content (post-industrial waste), the MFI increase due to the plasticizer in the NC binder was counteracted by non-volatile degradation products. Overall, the NC binder resin reduced stiffness and increased the elasticity of the recycled LDPE. The PU binder was degraded in the presence of moisture, resulting in an increased MFI and reduced mechanical properties. The PVB binder demonstrated thermal stability during mechanical recycling and acted as an impact modifier, improving the toughness of the recycled LDPE. The CAP binder had minimal impact on LDPE at the concentrations tested.

### 3.4. Limitations in the Experimental Study

After conducting the experimental measurements, the following limitations are recognized:

(a) The samples used in this study were non-pigmented, clear binder resins, allowing the impact of the binders to be isolated from that of pigments. A wide variety of pigments are used in printing inks, which poses a challenge in selecting a representative pigment sample matrix. As pigments are also commonly used to color plastics, it is recommended that the same pigments are used for both printing inks and plastic coloration to minimize any potential influence. Recommendations for pigment selection can be found in the work of Guo et al. 2024 [36].
(b) Some of the 3 wt.% moisture introduced to simulate the residual humidity in the input of extruder may have been lost due to evaporation. Consequently, the water content involved in the extrusion and degradation process was possibly lower than 3 wt.%. Nevertheless, the experimental results have shown that the moisture content is still sufficient for facilitating hydrolysis degradation. As the value of 3 wt.% is reported in literature after the washing and drying process in industrial operation [37], varying this amount in the experimental design is not essential.

## 4. Implications for Packaging Design and Recycling Process

In order to promote the circularity of flexible plastic packaging material, notably LDPE, it is essential to consider both plastic packaging design and the recycling process to minimize the negative impact due to printing ink on the quality of the recycled plastic.

Currently, the majority of single-layer, flexible plastic packaging uses NC-based printing ink. Previous studies have shown that NC binders can be effectively de-inked at relatively low temperatures (55 °C) [58]. In contrast, PU and PVB binders require special de-inking processes involving organic solvents (such as formic acid) or high temperatures (70–80 °C) and high alkalinity (2–5 wt.% NaOH) [12,28]. The findings of this study suggest that PU can be degraded by hydrolysis promoted by the residual moisture generated in the washing process upstream of extrusion. The degradation products were not detected by the GC-MS analysis that considered semi-volatile compounds. Therefore, the degradation products should be considered as volatile organic compounds (VOCs), confirmed in a recent study [16]. The VOCs in recycled plastic can be significantly reduced by degassing in the extrusion process and further reduced by employing a deodorizer following regranulation [59,60]. However, the pigments in the printing ink will still remain in the polymer matrix.

As a result of the C-C backbone in the PVB binder, partial degradation by hydrolysis (Figure 2d) can potentially generate products that are similar to those resulting from LDPE C-C chain oxidation [61]. The quality of recycled plastic from plastic packaging printed with a low content of PU or PVB binder (1 wt.%, corresponding to approximately 2 wt.% printing ink) is not significantly affected, and only the pigments remain in the polymer matrix.

Current optical sorting technology can differentiate colored and noncolored plastic packaging, regardless of printing, by identifying the spectral mix of thin layers [62,63]. Future developments should consider the fractionation of different printing ink binders for specific plastic packaging types. Single-layer, clear LDPE film may continue to use NC binders, provided that de-inking processes are available in recycling plants to maintain the transparency and light color of recycled material. In the case of colored, single-layer LDPE films, PU and PVB binders are preferable, with a printing ink content limited to 1 wt.% binder. CAP binders, which cannot be effectively de-inked or degraded during extrusion, exhibit a relatively minor impact due to their limited use as barrier layers in food packaging.

Multilayer plastic packaging represents an important flexible plastic packaging stream that does not currently undergo effective mechanical recycling [64,65]. PU is the major printing ink binder used in this type of packaging. In addition, the adhesion between different layers is PU-based. The results generated in this study regarding PU may provide a potential solution in designing more recyclable multilayer plastic packaging with mono-material (i.e., only PP or LDPE), if a recyclable compatible barrier layer (i.e., EVOH) is used [66,67].

## 5. Conclusions

This study has examined the degradation of four common printing ink binder resins (nitrocellulose (NC), polyurethane (PU), polyvinyl butyral (PVB), and cellulose acetate propionate (CAP)) under simulated recycling conditions that includes residual moisture from prewashing. The results have revealed that these different binders degrade differently from each other. The NC binders were degraded during extrusion, consistent with prior findings, and the presence of plasticizers led to an increased melt–flow index (MFI) and reduced stiffness. The non-volatile degradation products of NC may act as fillers, modifying the mechanical properties. The PU binders degraded only hydrolytically, as confirmed by FTIR and TGA analysis, but this resulted in a lesser impact on LDPE quality. PVB, due to its low melt viscosity, increased the MFI and acted as a toughening agent, enhancing stiffness in the recycled LDPE. Minimal side chain degradation was observed. The CAP binders exhibited stability under the extrusion conditions, with consequent negligible effects on the recycled material due to their low concentration in the waste stream.

The findings of this study can inform the design and recycling of flexible plastic packaging. NC-based inks may be efficiently recycled if de-inking systems are implemented in recycling plants. PU and PVB binders show minimal impact on LDPE quality at low concentrations, enabling them to be suitable for colored films, where de-inking is less critical. The insights offered regarding PU hydrolytic degradation open opportunities for developing recyclable multilayer packaging, which warrants further exploration. Future work should focus on refining recycling strategies tailored to binder types and optimizing packaging designs that facilitate recycling and enhance material quality.

## Figures and Tables

**Table 1:** Advantages and disadvantages of the printing ink binder resins investigated in this study, with associated thermal stability from TGA data reported in the literature; the asterisk denotes information obtained from interviewing printing ink experts.

Printing Ink Binder Resin	Advantages	Disadvantages	Thermal Stability
Nitrocellulose (NC)	Low price and high gloss [12,16]	Low thermal stability forming gas and brownish color in mechanical recycling process [26]	140–190 °C [14,27]
Polyurethane (PU)	Higher wear resistance and gloss [28] various formulations possible, depending on the constituent monomers [29]	Still uncommon for flexographic printing (single layer) due to adhesion [12]	Aliphatic PUs stable up to 250 °C [30,31]
Polyvinyl butyral (PVB)	High thermal stability and resistance [23]	May cause problem in sealing *	Stable up to 260 °C [20]
Cellulose acetate propionate (CAP)	Barrier for protecting other ink layer(s) *	Only limited to certain food packaging applications *	300–400 °C [25,32]

**Table 2:** (a). Binder–LDPE 5/95 blends prepared through solvent blending and extrusion under dry and wet conditions to compare degradation behavior. (b) Sample matrix prepared to mimic industrial recycling processes, investigating the impact of various binder contents on recyclate quality (* blank represents virgin LDPE).

**(a)**			
**Sample Name**	**Binder (wt.%)**	**LDPE (wt.%)**	**Preparing Method**
NC-P	100	0	Pure binder resin
NC-5S	95	5	Solvent blended
NC-5D	95	5	Extruded, dry condition
NC-5	95	5	Extruded, with 3 wt.% humidity
PU-P	100	0	Pure binder resin
PU-5S	95	5	Solvent blended
PU-5D	95	5	Extruded, dry condition
PU-5	95	5	Extruded, with 3 wt.% humidity
PVB-P	100	0	Pure binder resin
PVB-5S	95	5	Solvent blended
PVB-5	95	5	Extruded, with 3 wt.% humidity
CAP-P	100	0	Pure binder resin
CAP-1S	95	5	Solvent blended
CAP-1	95	5	Extruded, with 3 wt.% humidity
**(b)**			
**Binder**	**Sample Label**		**Binder content (wt.%)**
NC	NC-1		1
	NC-3		3
	NC-5		5
PU	PU-1		1
	PU-3		3
	PU-5		5
PVB	PVB-1		1
	PVB-3		3
	PVB-5		5
CAP	CAP-0.5		0.5
	CAP-1		1
Blank *	Blank dry (extruded)		0

**Table 3:** Comparison of different parameters associated with the blank samples extruded with and without 3 wt.% moisture.

Quality Parameter	Blank Dry (Without Moisture)	Blank Wet (With Moisture)	*p*-Value	Statistical Difference
MFI (g/10 min@ 190 °C, 2.16 kg)	0.68 ± 0.12	0.75 ± 0.08	0.45	No
Young’s modulus (MPa)	280.88 ± 2.18	279.83 ± 3.00	0.51	No
Tensile strength (MPa)	14.52 ± 0.17	14.70 ± 0.23	0.15	No
Strain at break (%)	40.43 ± 1.38	42.08 ± 1.18	0.05	No
Crystallinity temperature (°C)	97.02 ± 0.45	97.56 ± 0.08	0.24	No
Melt temperature (°C)	110.77 ± 0.47	109.87 ± 0.06	0.12	No
Degree of crystallinity (%)	20.73 ± 0.55	19.41 ± 0.19	0.09	No

**Table 4:** Overall impact of printing ink binder on the quality of recycled LDPE.

	Impact on Recycled LDPE		
Printing Ink Binder	MFI	Mechanical Properties	Thermal Properties
NC	Increased due to plasticizer; the non-volatile degradation product decreased the MFI	1 wt.% NC showed no impact on mechanical property; at higher content, the elasticity was increased	No impact on melt temperature, crystallization temperature, or degree of crystallinity in all samples (Supplementary Material S3.3)
PU	1 wt.% PU showed no impact; higher content can increase the MFI	Decreased both tensile strength and elasticity	
PVB	1 wt.% PVB showed no impact; higher content can increase the MFI	Increased Young’s modulus	
CAP	No impact	Minor impact	

## Data Availability

The Supplementary Material provides detailed data from the experimental part and further inquiry regarding the data upon requirement.

## Supplementary Materials

The following supporting information can be downloaded at: https://www.mdpi.com/article/10.3390/polym16233234/s1, Table S1.1. Exemplary composition of printing inks; Table S2.1. Composition of Printing Ink Binder Samples used in this Experiment; Table S2.2. Sample matrix for extrusion blends; Table S2.3. Sample matrix for solvent blends; Table S3.1. Detected substances from GC-MS analysis; Table S3.2. DSC results of all the extrusion samples including crystallinity temperature (Tc), melt temperature (Tm) and degree of crystallinity (Χc); Figure S2.1. Extruder screw configuration and temperature settings; Figure S2.2. Preparation procedure for the extrusion samples; Figure S3.1. Stress-strain curved of the extrusion blends, averaged by six replications of each sample; Figure S3.2. Result of GC-MS of total semi-volatile components. References [29,50,68,69,70,71,72,73,74,75,76] are cited in the supplementary materials.
