# Supplementary material for: Overlooked Impact of Moisture on the Stability of Printing Ink and Its Impact on Recycled Low-Density Polyethylene (LDPE) Quality
Source: Polymers (Basel). 2024 Nov 21;16(23):3234. doi: 10.3390/polym16233234 (PMC11644606; doi:10.3390/polym16233234)
Supplement: Supplementary file 1 [file polymers-16-03234-s001.zip › polymers-3243032-supplementary.pdf]

Supplementary Material

Overlooked Impact of Moisture on the Stability of Printing Ink and its Impact on Recycled Low-Density Polyethylene (LDPE) Quality

Jinyang Guo* , Willi Wagner, Iryna Atamaniuk , Zhi Kai Chong , Ayah Alassali , Kerstin Kuchta

Hamburg University of Technology (TUHH)

Circular Resource Engineering and Management (CREM)

Blohm Strasse 15, 21079 Hamburg

Supplementary Material S1: Exemplary Compositions of Printing Inks for Plastic Packaging

Table S1.1. Exemplary composition of printing inks

**Exemplary composition 1 [68]**	**Concentration (wt.%)**
Titanium dioxide	35.00
Nitrocellulose	5.00
Polyamide	15.00
Dibutyl phthalate (plasticizer)	1.00
Polyethylene wax	1.00
ethanol	30.00
n-propyl acetate	8.00
n-propanol	4.00

**Exemplary composition 2 [69]**	**Concentration (wt.%)**
Polymeric plasticizer	5.40
Monomeric plasticizer	2.00
Pigment PB 15:4	1.1
Pigment PR 57:1	2.1
Pigment PY 13	2.2
Pigment PBk 7	1.00

Nitrocellulose	21.00
Wax	0.40
Adhesion promoter	1.5
Solvent	63.3

**Exemplary composition 3 [69]**	**Concentration (wt.%)**
Aliphatic polyurethane	29.50
Pigment PB 15:4	1.00
Pigment PR 57:1	1.90
Pigment PY 13	1.90
Pigment PBk 7	1.00
Inorganic filler	2.70
Wax	0.20
Solvent	61.90

**Exemplary composition 4*[70]**	**Concentration (wt.%)**
Organic pigment	12.00
Alcohol-soluble polyamide	22.00
Nitrocellulose	4.0
Ethanol	29.00
n-propyl alcohol	18.00
n-propyl acetate	10.00
Polyethylene wax	4.0
Fatty acid amide	1.0

* This printing ink formulation is old, the application of polyamide does still exist today, but with a relatively low share on the modern ink market

Supplementary Material S2: Sample Preparation

S2.1 Printing ink binder

All the printing ink binders were delivered in solution form, an overview of the dissolved binders and their net binder content in wt.% are shown in Table S2.1.

Table S2.1. Composition of Printing Ink Binder Samples used in this Experiment

Sample	Organic solution	Solid content (wt.%)
Nitrocellulose (NC)	Ethanol	19.55
Polyurethane (PU)	Ethyl acetate	38.00
Polyvinyl butyral (PVB)	Ethanol	20.26
Cellulose acetate propionate (CAP)	Ethyl acetate	20.34

S2.2 Extrusion sample preparing

The screw configuration of the extruder is shown in Figure S2.1 and the preparation procedure for the extrusion samples simulating printing ink is shown in Figure S2.2.

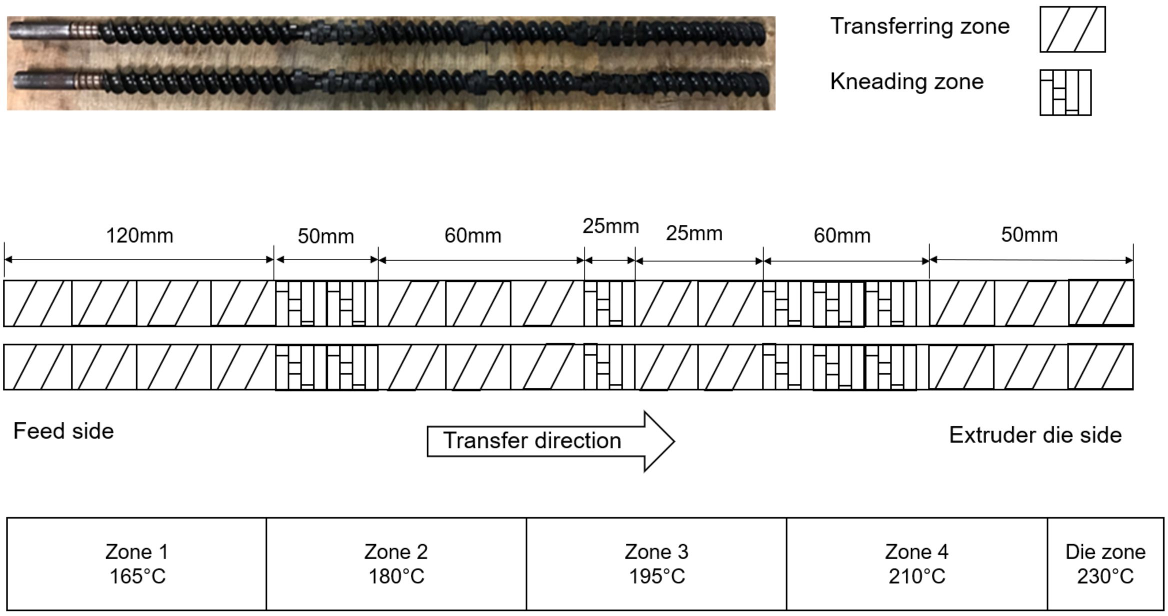

Figure S2.1. Extruder screw configuration and temperature settings.

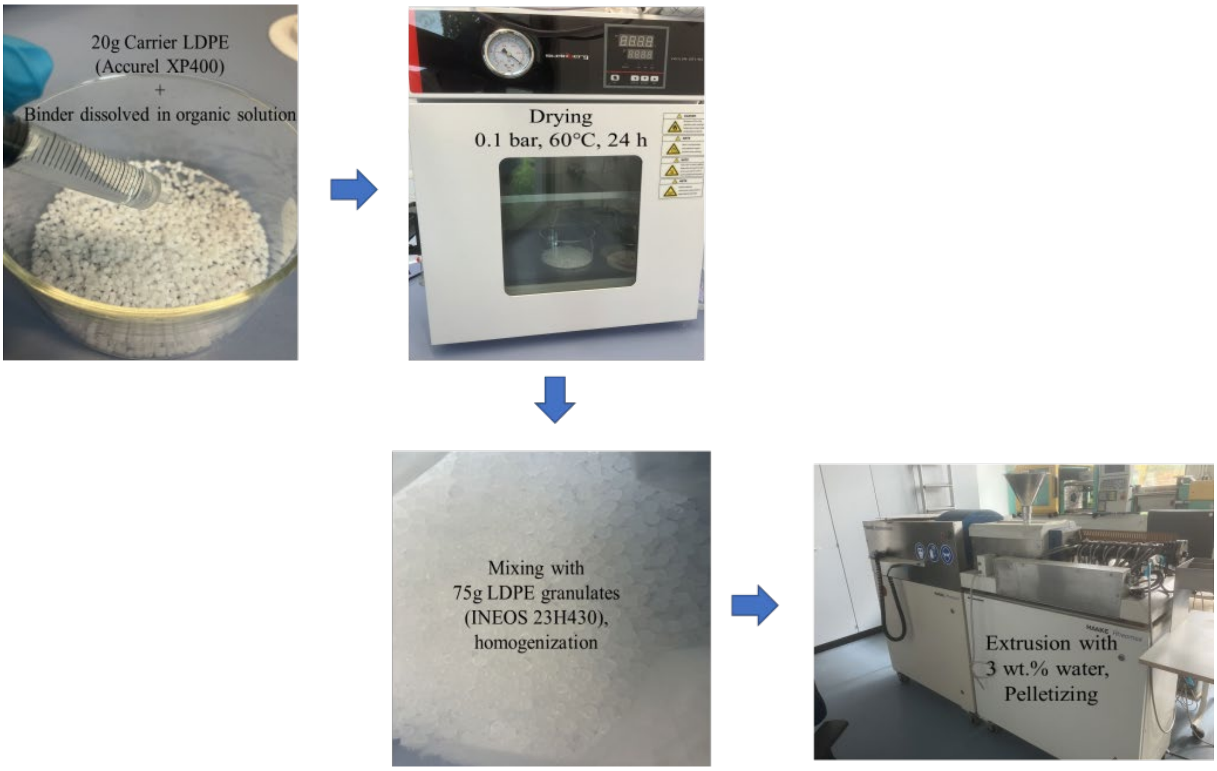

Figure S2.2 . Preparation procedure for the extrusion samples

The sample matrix for extrusion is shown in Table S2.2

Table S2.2. Sample matrix for extrusion blends

Sample name	Base material	Binder name	Binder dry content (wt.%)*	Water added (wt.%)
Blank dry	20g Carrier LDPE (Evonik Accurel XP400) and 75g film grade LDPE granulates (INEOS 23H430)	none	0	0
Blank wet			0	3
NC-1		Nitrocellulose	1	3
NC-3			3	3
NC-5			5	3
PU-1		Polyurethane	1	3
PU-3			3	3
PU-5			5	3
PVB-1		Polyvinyl butyral	1	3
PVB-3			3	3
PVB-5			5	3
CAP-0.5		Cellulose acetate propionate	0.5	3
CAP-1			1	3

S2.3 Dissolution sample preparing

The sample matrix for blending polymers with dissolution process is shown in Table S2.3.

Table S2.3. Sample matrix for solvent blends

Sample name	Base material	Binder name	Binder dry content (wt.%)*
Virgin S	20g Carrier LDPE (Evonik Accurel XP400) and 75g film grade LDPE granulates (INEOS 23H430)	None	0
NC-S		Nitrocellulose	5
PU-S		Polyurethane	5
PVB-S		Polyvinyl Butyral	5
CAP-S		Cellulose Acetate Propionate	1

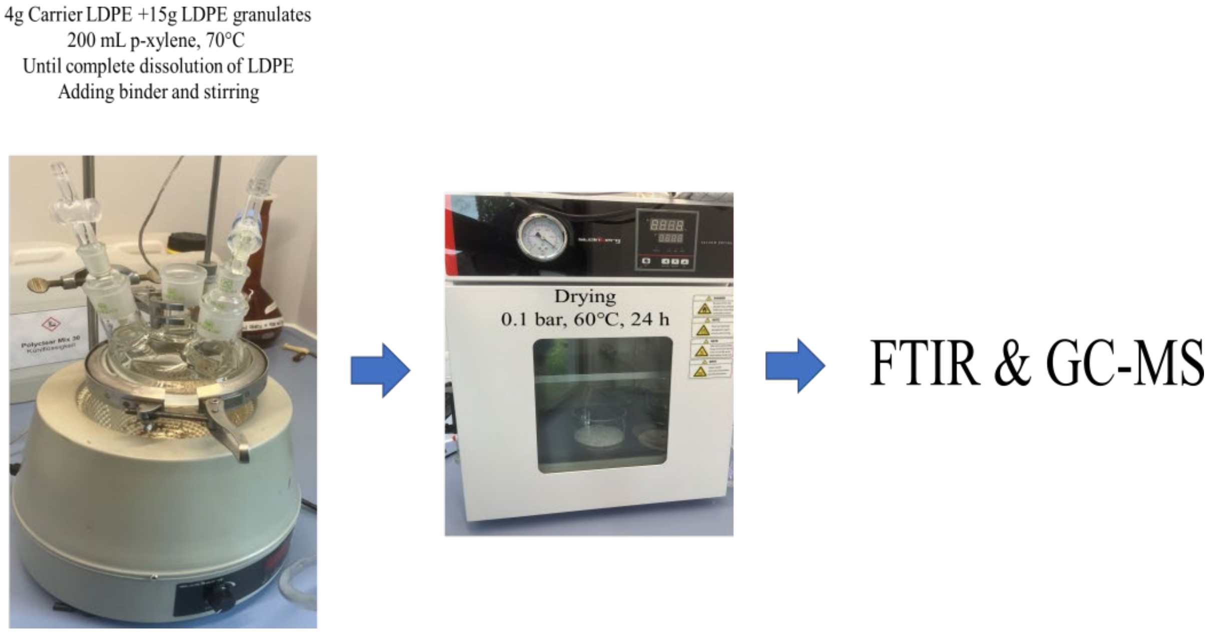

Figure S2.3. Preparation procedure for the solvent blend samples

Supplementary Material S3: Detailed Analysis Results

This part of the supplementary material presents the detailed analysis results including tensile strength, GC-MS and DSC.

S3.1 Detailed stress-strain curve of tensile strength test

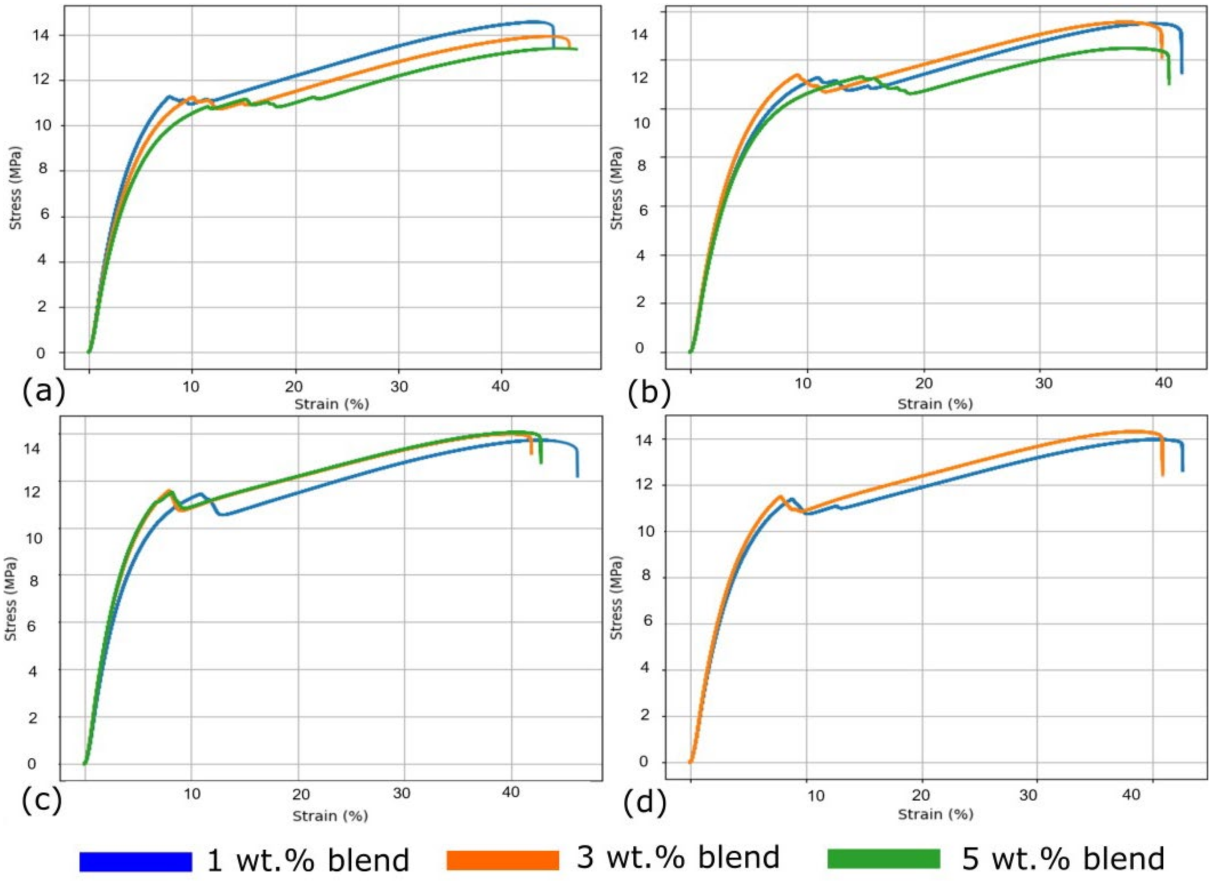

Figure S3.1. Stress-strain curved of the extrusion blends, averaged by six replications of each sample.

Table 1. Tensile strength properties including Young's modulus, tensile strength, and strain at break of all the extruded samples

Sample Name	Young's Modulus (MPa)	Tensile Strength (MPa)	Strain at break (%)
Blank dry	280.88±2.18	14.32±0.17	40.43±1.28
Blank wet	279.83±3.00	14.7±0.23	42.08±1.18
NC-1	277.83±9.07	14.59±0.60	43.73±1.40
NC-3	254.44±6.08	13.95±0.26	45.07±1.04
NC-5	239.40±13.08	13.43±0.36	46.18±1.83
PU-1	271.87±5.75	13.50±0.23	39.51±1.06
PU-3	261.50±5.21	13.55±0.15	37.31±0.55
PU-5	251.02±4.60	12.48±0.13	37.68±0.90
PVB-1	266.49±2.30	13.71±0.14	42.74±0.66
PVB-3	295.10±3.03	13.98±0.14	40.27±1.00
PVB-5	299.20±7.67	14.06±0.58	40.88±1.20
CAP-0.5	275.53±8.42	13.98±0.23	40.43±0.72
CAP-1	288.41±2.89	14.33±0.41	38.64±1.30

S3.2 Result from the GC-MS analysis

All the results were blank subtracted. The subtracted black samples including the virgin LDPE (blank dry and blank wet for extruded samples, Virgin-S for solvent blended samples), P-xylene for preparing the solvent blend and ethanol used for the extraction. The cumulative results of each sample is shown in Figure S3.2, the list of detect substances of the analyzed samples is shown in Table

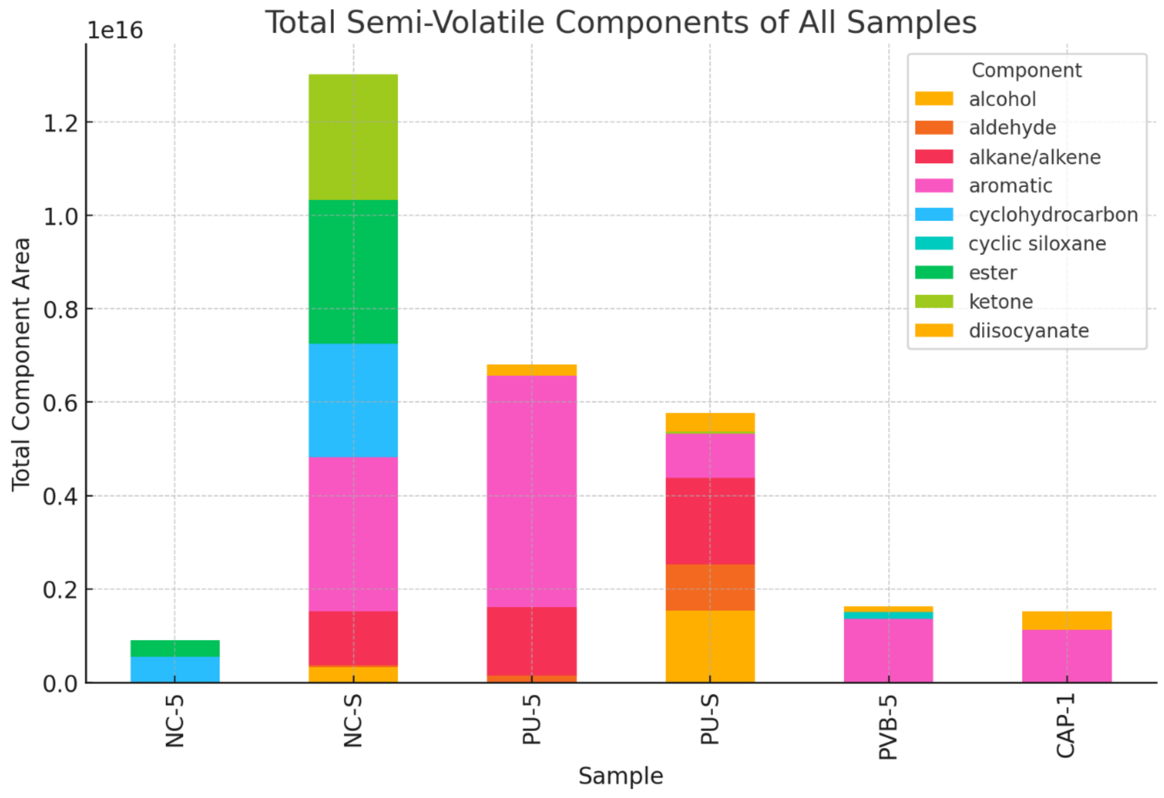

Figure S3.2. Result of GC-MS of total semi-volatile components

Table S3.1. Detected substances from GC-MS analysis

NC-5	Compound Name	Component Area	Chemical family	Remarks
	1,4:3,6-Dianhydro-.alpha.-d-glucopyranose	2.888E+14	cyclic ether	biomass degradation product [71]
	n-Tridecylcyclohexane	2.65437E+14	cycloalkane	
	Tributyl acetylcitrate	1.15651E+14	ester	plasticizer [50]
	Hexanedioic acid, bis(2-ethylhexyl) ester	2.3121E+14	ester	plasticizer [72]
**NC-S**	**Compound Name**	**Component Area**	**Chemical family**	**Remarks**
	1-Octadecanol	3.30568E+14	alcohol	
	Benzaldehyde	4.4099E+13	aldehyde	
	Undecane	2.46862E+14	alkane	
	Dodecane, 4,9-dipropyl-	1.49892E+14	alkane	
	1-Heptadecene	3.89755E+14	alkene	
	1-Nonadecene	3.55855E+14	alkene	
	Benzyl chloride	5.90121E+14	aromatic	
	Benzene, 1-methyl-3-(1-methylethyl)-	2.39021E+14	aromatic	
	Butylated Hydroxytoluene	6.65894E+14	aromatic	antioxidant [73]
	Benzene, (1-pentylheptyl)-	1.39505E+14	aromatic	
	Benzene, (1-butyloctyl)-	1.31009E+14	aromatic	
	Phenol, 2,6-bis(1,1-dimethylethyl)-4-ethyl-	1.38871E+14	aromatic	antioxidant [73]
	Benzene, (1-ethyldecyl)-	1.76338E+12	aromatic	
	Benzene, (1-methylundecyl)-	5.34987E+14	aromatic	
	2-(Diethylamino)ethyl 4-amino-2-hydroxybenzoate	8.70042E+14	aromatic amine	
	1,4:3,6-Dianhydro-.alpha.-d-glucopyranose	6.44485E+14	cyclic ether	biomass degradation product [74]
	Levoglucosenone	7.46326E+14	cyclic ether	biomass degradation product [74]

	Compound Name	Component Area	Chemical family	Remarks
	2,6-Di-tert-butyl-4-hydroxy-4-methylcyclohexa-2,5-dien-1-one	2.83797E+14	cyclic ketone	
	n-Tridecylcyclohexane	3.82408E+14	cycloalkane	
	Bicyclo[3.1.1]hept-2-en-6-one, 2,7,7-trimethyl-	1.51674E+14	cycloalkene	
	Cyclohexane, undecyl-	2.1563E+14	cycloalkene	
	Ethyl acetoacetate	5.67149E+14	ester	
	Diethyl malonate	9.66609E+14	ester	
	Tributyl acetylcitrate	5.87649E+14	ester	plasticizer [50]
	Hexanedioic acid, bis(2-ethylhexyl) ester	9.57512E+14	ester	plasticizer [72]
	2(5H)-Furanone	1.46422E+14	Ketone	
	1,2-Cyclopentanedione	2.17234E+14	Ketone	
	2H-Pyran-2,6(3H)-dione	1.71264E+14	Ketone	
**PU-5**	**Compound Name**	**Component Area**	**Chemical family**	**Remarks**
	Benzaldehyde	1.41524E+14	aldehyde	
	Dodecane, 2-cyclohexyl-	9.40478E+14	alkane	
	1-Heptadecene	5.30103E+14	alkene	
	p-Xylene	160182417	aromatic	contamination
	Benzoic acid	1.09069E+14	aromatic	raw material for printing ink [29]
	Butylated Hydroxytoluene	1.11138E+14	aromatic	antioxidant [73]
	Benzene, (1-propyloctyl)-	4.72601E+14	aromatic	
	Benzene, (1-pentylheptyl)-	6.67876E+14	aromatic	
	Benzene, (1-butyloctyl)-	6.65006E+14	aromatic	
	Isophorone diisocyanate	2.33548E+14	diisocyanate	raw material for printing ink [75]
**PU-S**	**Compound Name**	**Component Area**	**Chemical family**	**Remarks**
	Benzenemethanol, 4-methyl-	7.88448E+14	alcohol	

	Compound Name	Component Area	Chemical family	Remarks
	4,8,12,16-tetraoxaeicosan-1-ol	7.50583E+14	alcohol	raw material for coating [76]
	Benzaldehyde, 3-methyl-	1.33886E+14	aldehyde	
	Dodecane	8.37748E+13	alkane	
	1-Heptadecene	3.1433E+13	alkene	
	Butylated Hydroxytoluene	7.07534E+14	aromatic	antioxidant [73]
	Isophorone diisocyanate	4.02075E+14	diisocyanate	raw material for printing ink [75]
	E-15-Heptadecenal	5.32518E+13	Ketone	
**PVB-5**	**Compound Name**	**Component Area**	**Chemical family**	**Remarks**
	Benzene, 1-methyl-3-(1-methylethyl)-	1.47274E+14	aromatic	
	D-Limonene	2.19353E+14	aromatic	
	Butylated Hydroxytoluene	1.39066E+14	aromatic	antioxidant[73]
	Benzene, (1-pentylheptyl)-	8.54124E+14	aromatic	
	Cyclotetrasiloxane, octamethyl-	1.51208E+14	cyclic siloxane	
	Isophorone diisocyanate	1.11916E+14	diisocyanate	
**CAP-1**	**Compound Name**	**Component Area**	**Chemical family**	**Remarks**
	D-Limonene	2.17538E+13	1.23434E+12	
	2,6-Dimethylphenyl isocyanate	3.90201E+14		
	Phenol, 4-(1,1-dimethylpropyl)-	1.26322E+14	3.63983E+14	
	Benzene, (1-pentylheptyl)-	9.81605E+14	2.95337E+14	

S3.3 Detailed DSC results

The DSC results is shown in Table S3.2, since not statistical difference, the results were not included in the results and discussion part of the manuscript.

Table S3.2. DSC results of all the extrusion samples including crystallinity temperature (Tc), melt temperature (Tm) and degree of crystallinity (Xc)

Sample name	Tc(°C)	STDEV	Tm (°C)	STDEV	Xc (%)	STDEV
Blank dry	97.02	0.45	110.77	0.47	20.73%	0.55%
Blank wet	97.56	0.08	109.87	0.06	19.41%	0.19%
NC-1	95.22	0.03	112.20	0.36	17.04%	1.25%
NC-3	95.85	0.57	111.77	0.37	18.44%	1.48%
NC-5	96.38	0.93	111.02	2.47	20.16%	4.42%
PU-1	97.00	0.11	110.43	0.06	18.83%	0.34%
PU-3	96.83	1.25	110.53	1.10	16.74%	1.61%
PU-5	95.52	0.46	111.84	0.81	17.31%	1.62%
PVB-1	97.23	0.11	109.97	0.20	17.27%	1.04%
PVB-3	96.45	0.01	110.81	0.38	17.90%	1.00%
PVB-5	96.03	0.34	112.26	0.65	18.21%	0.45%
CAP-0.5	96.91	1.41	110.48	1.55	16.91%	0.91%
CAP-1	97.02	0.32	110.46	0.18	17.89%	0.68%
